# Relationship of tumor PD‐L1 (CD274) expression with lower mortality in lung high‐grade neuroendocrine tumor

**DOI:** 10.1002/cam4.1172

**Published:** 2017-09-18

**Authors:** Kentaro Inamura, Yusuke Yokouchi, Maki Kobayashi, Hironori Ninomiya, Rie Sakakibara, Makoto Nishio, Sakae Okumura, Yuichi Ishikawa

**Affiliations:** ^1^ Division of Pathology The Cancer Institute Department of Pathology The Cancer Institute Hospital Japanese Foundation for Cancer Research 3‐8‐31 Ariake, Koto‐ku Tokyo 135‐8550 Japan; ^2^ Translational Medicine & Clinical Pharmacology Department Daiichi Sankyo Co., Ltd. 1‐2‐58, Hiromachi Shinagawa‐ku Tokyo 140‐0005 Japan; ^3^ Department of Integrated Pulmonology Tokyo Medical and Dental University 1‐5‐45, Yushima Bunkyo‐ku Tokyo 113‐8519 Japan; ^4^ Department of Thoracic Medical Oncology The Cancer Institute Hospital Japanese Foundation for Cancer Research 3‐8‐31 Ariake, Koto‐ku Tokyo 135‐8550 Japan; ^5^ Department of Thoracic Surgical Oncology The Cancer Institute Hospital Japanese Foundation for Cancer Research 3‐8‐31 Ariake Koto‐ku Tokyo 135‐8550 Japan

**Keywords:** Immune checkpoint, LCNEC, lung cancer, outcome, PD‐L1, small cell carcinoma

## Abstract

Programmed death‐ligand 1 (PD‐L1) promotes immunosuppression by binding to PD‐1 on T lymphocytes. Although tumor PD‐L1 expression is a potential predictive marker of clinical response to anti‐PD‐1/PD‐L1 therapy, little is known about its association with clinicopathological features, including prognosis, in high‐grade neuroendocrine tumors (HGNETs), including small‐cell lung carcinoma (SCLC) and large‐cell neuroendocrine carcinoma (LCNEC), of the lung. We immunohistochemically examined the membranous of expression of PD‐L1 in 115 consecutive surgical cases of lung HGNET (74 SCLC cases and 41 LCNEC cases). We examined the prognostic association of tumor PD‐L1 positivity using the log‐rank test as well as Cox proportional hazards regression models to calculate the hazard ratio (HR) for mortality. Programmed death‐ligand 1 immunostaining (at least 5% tumor cells) was observed in 25 (21%) of the 115 HGNET cases. In a univariable analysis, PD‐L1 positivity was associated with lower lung cancer‐specific (univariable HR = 0.23; 95% confidence interval [CI] = 0.056–0.64; *P *=* *0.0028) and overall (univariable HR = 0.28; 95% CI = 0.11–0.60; *P *=* *0.0005) mortality. Additionally, in a multivariable analysis, PD‐L1 positivity was independently associated with lower lung cancer‐specific (multivariable HR = 0.24; 95% CI = 0.058–0.67; *P *=* *0.0039) and overall (multivariable HR = 0.29; 95% CI = 0.11–0.61; *P *=* *0.0006) mortality. Our study demonstrated the prevalence of PD‐L1 positivity in lung HGNET cases, and the independent association of tumor PD‐L1 positivity with lower mortality in lung HGNET cases. Further studies are needed to confirm our findings.

## Introduction

High‐grade neuroendocrine tumors (HGNETs), comprising small cell lung carcinoma (SCLC) and large cell neuroendocrine carcinoma (LCNEC), constitute the most aggressive lung cancer histological subtype 1. SCLCs and LCNECs have common and distinct clinicopathological features characterized by poorer prognosis than non‐SCLC (NSCLC; except LCNEC), neuroendocrine differentiation, and a heavy smoking history, which results in a higher mutation burden, more tumor antigens, and higher immunogenicity 2, 3, 4, 5.

Programmed death‐ligand 1 (PD‐L1 or CD274), an immune modulator, promotes immunosuppression by binding to programmed death‐1 (PD‐1 or PDCD1) on T lymphocytes. Emerging evidence suggests that anti‐PD‐1/PD‐L1 therapy is effective in various malignancies, including SCLC, and PD‐L1 expression in cancer cells is a potential predictive marker of the clinical response to anti‐PD‐1/PD‐L1 therapy 6, 7, 8, 9, 10, 11, 12, 13, 14, 15. Interestingly, the response to anti‐PD‐1/PD‐L1 therapy for lung cancer treatment is better in smokers than in nonsmokers 16, 17, 18, 19.

To our knowledge, no study has examined the association of tumor PD‐L1 expression with prognosis in HGNET, although a few studies have examined PD‐L1 expression in SCLC with conflicting results 20, 21, 22. Therefore, we set out a study to analyze expression of PD‐L1 protein using a monoclonal antibody and investigate if there was any association with survival. First, we validated specificity of the antibody, size of the protein, and a relation of expression between gene and protein, employing cultured cell lines. Then, as a primary objective, we examined the association of the protein expression with prognosis of lung HGNET via 115 consecutive cases, comprising 74 and 41 cases of SCLC and LCNEC, respectively.

## Materials and Methods

### Study population

We examined 115 consecutive lung HGNET cases, including 74 SCLC and 41 LCNEC cases, to assess the prognostic association of membranous PD‐L1 expression in HGNET 23. These cases underwent surgical resections between July 1990 and November 2014 at The Cancer Institute Hospital, Japanese Foundation for Cancer Research (JFCR) in Tokyo, Japan. Patients were followed up until May 1, 2016 or death, whichever was earlier. Smoking exposure was measured as pack‐years by multiplying the “number of cigarette packs per day” by “duration in years.” All patients provided informed consent for research, and the study plan was approved by the institutional review board of JFCR.

### Pathological evaluation

Pathological diagnoses were made by experienced expert pulmonary pathologists (KI and YI) according to the 2015 WHO classification of lung tumors 24. All patients were pathologically staged according to the AJCC‐TNM staging system, 7th edition 25.

### Immunohistochemistry for PD‐L1

Membranous PD‐L1 expression of cancer cells was evaluated by tissue microarray immunohistochemical analysis. Using archived surgically resected specimens, which had been used for the initial pathological diagnosis of primary lung cancer, we constructed tissue microarrays as described previously 26. In brief, we punched points on the donor paraffin blocks using a 2‐mm‐diameter coring needle and transferred the tissue to the array in the recipient block using a manual tissue arrayer (KIN; Azumaya, Tokyo, Japan). For each tumor, an experienced pulmonary pathologist (KI) selected one 2‐mm‐diameter site showing the tumor's most representative histology 27.

**Figure 1:**
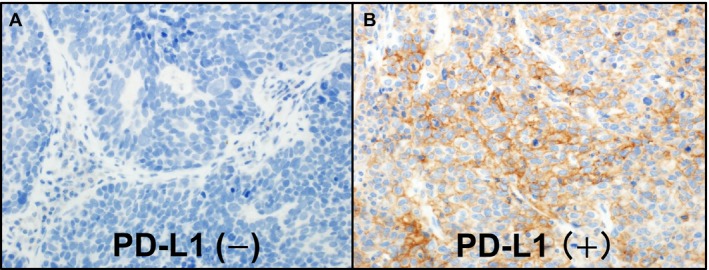
Immunohistochemical evaluation of tumor membranous programmed death‐ligand 1 (PD‐L1) expression in small cell lung carcinoma. (A) PD‐L1 negative, (B) PD‐L1 positive.

Programmed death‐ligand 1 immunostaining was conducted as previously described 28. Sections of 4‐*μ*m thickness were immunostained for PD‐L1 with an anti‐PD‐L1 rabbit monoclonal antibody (clone: E1L3N; Cell Signaling Technology, Danvers, MA; diluted 1:50) using the Leica Bond III automated system (Leica Biosystems Melbourne Pty Ltd., Melbourne, Australia). Sections were incubated at pH 9 for 20 min at 100°C. Antibody‐based PD‐L1 expression was interpreted by a pathologist (KI) who was blinded to patient details. We used the same PD‐L1‐positive/‐negative criteria as those that were previously used 28 and validated in numerous studies 10, 20, 22, 29, 30. The tumor cell percentage with membranous PD‐L1 staining was recorded. Scores of <5% were categorized as “PD‐L1 negative” (Fig. 1A); those of ≥5% were categorized as “PD‐L1 positive” (Fig. 1B). All the 115 cases were evaluated by a second pathologist (YY) who was also blinded to patient details. The agreement between the two pathologists for PD‐L1 positivity was good with a kappa value of 0.64 (95% confidence interval [CI] = 0.44–0.83, *P *<* *0.0001), indicating substantial agreement.

We validated the specificity of the PD‐L1 antibody (clone: E1L3N). Because PD‐L1 is also known as B7‐H1, a B7 subfamily member, we used a B7 subfamily cell array (provided by Daiichi Sankyo Co., Ltd., Tokyo, Japan), comprising CHO‐K1 cells overexpressing the B7 subfamily. With 10% neutral buffered formalin, we fixed CHO‐K1 cells transiently overexpressing PD‐L1 (B7‐H1), B7‐H2, B7‐DC (PD‐L2), B7‐H3, B7‐H4, B7‐1, or B7‐2, and mock‐transfected control cells and processed them to yield a formalin‐fixed paraffin‐embedded (FFPE) cell block array. Using this B7 subfamily cell array, we assessed the specificity of the PD‐L1 antibody (clone: E1L3N). Furthermore, we used sections processed with primary antibody replacement by Dako REAL^™^ Antibody Diluent (Dako, Glostrup, Denmark) as nonspecific negative controls.

### Cell culture

Human SCLC cell lines, STC‐1 and Lu135, were obtained from Japanese Collection of Research Biosources (JCRB, Ibaraki, Japan). Cells were cultured in RPMI‐1640 (Gibco, Gran Island, NY) supplemented with 10% FBS at 37°C in a humidified atmosphere of 5% CO_2_ in the air.

### Western blot for PD‐L1

Total protein was extracted from STC‐1 and Lu135 cell lysate. Protein concentration was determined using BCA assay reagents (TAKARA, Tokyo, Japan), and 20 *μ*g protein samples were loaded into 4–12% Bis‐Tris gel (Invitrogen, Carlsbad, CA). After electrophoresis, proteins were transferred to polyvinylidene fluoride (PVDF) membrane (Invitrogen). The membrane was blocked by 5% skim milk (Nacalai Tesque, Kyoto, Japan) prior to incubation with rabbit monoclonal antibodies, anti‐PD‐L1‐antibody (clone: E1L3N, Cell Signaling Technology; diluted 1∶1000), and anti‐*β*‐actin‐antibody (clone: #4967, Cell Signaling Technology; diluted 1:3000), for 1 h at room temperature. Immunoblots were detected using SuperSignal West Femto kit (Thermo Scientific, Rockford, IL).

### RT‐PCR for PD‐L1

Total RNA was extracted from SCLC cells using RNeasy Mini kit (Qiagen, West Sussex, UK). For each sample, 100 ng of total RNA was reverse transcribed into cDNA using SuperScript III First Strand (Invitrogen) according to the manufacturer's instructions. The PD‐L1 gene was amplified with KOD FX neo (Toyobo, Osaka, Japan) using the following primers: 5′‐ATGGTGGTGCCGACTACAAG‐3′ and 5′‐GGAATTGGTGGTGGTGGTCT‐3′. We used PCR primers 5′‐CCAACTGGGACGACATGGAG‐3′ and 5′‐TGGATGGCTACGTACATGGC‐3′ for *β*‐actin.

The PCR conditions for PD‐L1 and *β*‐actin were as follows: 94°C for 2 min followed by 35 cycles (98°C for 10 sec, 61°C for 30 sec, and 68°C for 15 sec), and a final holding temperature of 4°C. After PCR, 5 *μ*L of each sample was electrophoresed on 2% agarose gel.

### Statistical analysis

We conducted all statistical analyses using JMP 12 (SAS Institute Inc., Cary, NC) and Excel 2016 (Microsoft, Redmond, WA). All *P*‐values were two sided, and we considered *P *=* *0.05 or less statistically significant. The primary objective of this study is to examine the association of tumor PD‐L1 expression with prognosis in patients with lung HGNET. All the other analyses were secondary and exploratory. We interpreted *P‐*values very cautiously to avoid overinterpretation by considering multiple‐hypothesis testing. To investigate the associations of tumor PD‐L1 positivity with clinicopathological factors in lung HGNET cases, we performed the chi‐square or Fisher's exact test as appropriate. We used the Kaplan–Meier method and log‐rank test for survival analyses. For analyses of lung cancer‐specific mortality, we censored deaths because of other causes. We used univariable and multivariable Cox proportional hazards regression models to calculate the hazard ratio (HR) for mortality according to PD‐L1 expression status (positive vs. negative). The multivariable model initially included age (<60 vs. ≥60 years), gender (male vs. female), smoking history (≤20 vs. >20 pack‐years), pathological stage (p‐stage) (I vs. II–IV), neoadjuvant chemotherapy (yes vs. no), adjuvant chemotherapy (yes vs. no), histology (SCLC vs. LCNEC), and lymphovascular invasion (positive vs. negative). We created missing categories for missing cases for each variable, if applicable, and performed a backward stepwise elimination with *P *=* *0.05 as the threshold to select variables for the final model. We confirmed the proportionality of hazards assumption using graphs of log(−log[survival probability]) versus log of survival time to visually assess if the lines were approximately parallel.

## Results

### Validation of specificity of the PD‐L1 antibody

We validated the specificity of the PD‐L1 antibody (clone: E1L3N) by using a B7 subfamily cell array, comprising CHO‐K1 cells transiently overexpressing PD‐L1 (B7‐H1), B7‐H2, B7‐DC (PD‐L2), B7‐H3, B7‐H4, B7‐1, or B7‐2, and mock‐transfected control cells. Using this cell array, we verified the specificity of the PD‐L1 antibody (clone: E1L3N) by confirming that this antibody exclusively recognized PD‐L1 (B7‐H1) among the tested B7 subfamilies (Figure S1).

We performed Western blotting and RT‐PCR analyses for PD‐L1, using the SCLC cell lines (STC‐1 and Lu135). The anti‐PD‐L1 antibody (clone: E1L3N) showed a PD‐L1 protein in Lu135, but not in STC‐1 (Figure S2a). The size of protein was identical to PD‐L1. Lu135 also showed a PD‐L1 transcript, whereas STC‐1 did not show a PD‐L1 transcript (Figure S2b). We confirmed that the PD‐L1 protein expression corresponded to the gene expression.

### Tumor PD‐L1 expression in lung HGNET

Among the 115 lung HGNET cases, we observed PD‐L1 positivity (≥5% tumor cells with membranous staining) in 25 cases (21%) by immunohistochemistry (Fig. 1). The clinicopathological characteristics of lung HGNET cases are summarized according to PD‐L1 expression status in Table 1. PD‐L1 positivity was associated with absence of neoadjuvant chemotherapy (*P *=* *0.041). However, of the 115 HGNEC patients, only 22 SCLC patients underwent neoadjuvant chemotherapy because many patients were in the early stage, and PD‐L1 positivity showed a tendency for the association of no neoadjuvant chemotherapy in SCLC (*P *=* *0.052).

**Table 1:** Clinicopathological characteristics of lung high‐grade neuroendocrine tumor (HGNET) according to tumor programmed death‐ligand 1 (PD‐L1) expression

Variables	*N* of samples (%)	PD‐L1 expression		
		Negative (*n* = 90)	Positive (*n* = 25)	*P*‐values
Age (years)				0.56
<60	28 (24%)	23 (26%)	5 (20%)	
≥60	87 (76%)	67 (74%)	20 (80%)	
Gender				0.21
Male	93 (81%)	75 (83%)	18 (72%)	
Female	22 (28%)	15 (17%)	7 (28%)	
Smoking history (pack‐years)				0.56
≤20	28 (24%)	23 (26%)	5 (20%)	
>20	87 (76%)	67 (74%)	20 (80%)	
Tumor size (mm)				0.65
≤30	69 (60%)	55 (61%)	14 (56%)	
>30	46 (40%)	35 (39%)	11 (44%)	
p‐stage				0.53
I	53 (46%)	40 (45%)	13 (52%)	
II–V	61 (53%)	49 (55%)	12 (48%)	
Neoadjuvant chemotherapy				0.041
No	93 (81%)	69 (77%)	24 (96%)	
Yes	22 (19%)	21 (23%)	1 (4.0%)	
Adjuvant chemotherapy				0.21
No	50 (43%)	40 (44%)	10 (40%)	
Yes	65 (57%)	50 (56%)	15 (60%)	
Histology				0.33
SCLC	74 (64%)	60 (67%)	14 (56%)	
LCNEC	41 (36%)	30 (33%)	11 (44%)	
Lymphovascular invasion				0.45
Negative	11 (9.7%)	10 (11%)	1 (4.0%)	
Positive	103 (90%)	79 (89%)	24 (96%)	

Percentage indicates the proportion of cases with a specific clinical, pathological, or molecular feature among each category. We performed the chi‐square test or Fisher's exact test as appropriate. HGNET, high‐grade neuroendocrine tumor; LCNEC, large cell neuroendocrine carcinoma; p‐stage, pathological stage; SCLC, small cell lung carcinoma.

### PD‐L1 positivity and survival of lung HGNET patients

**Figure 2:**
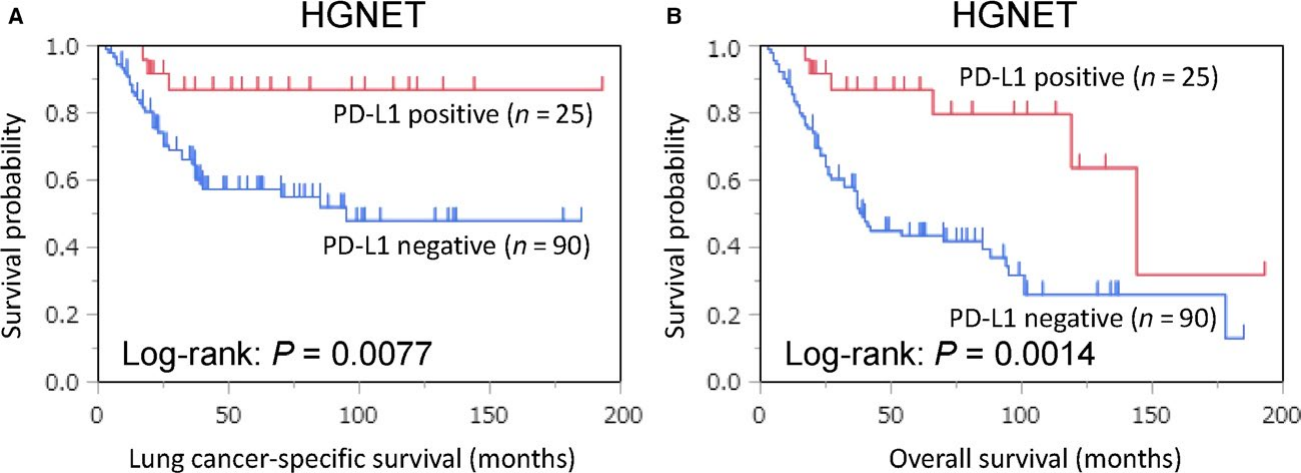
Kaplan–Meier curves for lung cancer‐specific (A) and overall survival (B) according to tumor programmed death‐ligand 1 (PD‐L1) expression status (positive vs. negative) in high‐grade neuroendocrine tumor (HGNET).

Among the 115 patients, there were 62 overall deaths, including 40 lung cancer‐specific deaths, during a median follow‐up of 5.8 years (interquartile range: 3.1–8.2 years) for those censored. PD‐L1 positivity was associated with lower lung cancer‐specific (log‐rank, *P *=* *0.0077) and overall (log‐rank, *P *=* *0.0014) mortality (Fig. 2). In univariable Cox regression analysis, PD‐L1 positivity was associated with lower lung cancer‐specific (univariable HR = 0.23; 95% CI=0.056–0.64; *P *=* *0.0028) and overall (univariable HR=0.28; 95% CI = 0.11–0.60; *P *=* *0.0005) mortality (Table 2). In multivariable Cox regression analysis, PD‐L1 positivity was independently associated with lower lung cancer‐specific (multivariable HR = 0.24; 95% CI = 0.058–0.67; *P *=* *0.0039) and overall (multivariable HR = 0.29; 95% CI = 0.11–0.61; *P* = 0.0006) mortality. Among the other covariates, p‐stage II–IV was associated with higher lung cancer‐specific (vs. p‐stage I; multivariable HR = 3.22; 95% CI = 1.64–6.82; *P *=* *0.0006) and overall (vs. p‐stage I; multivariable HR = 2.07; 95% CI = 1.23–3.56; *P *=* *0.0062) mortality (Table 2).

**Table 2:** Programmed death‐ligand 1 (PD‐L1) expression and other covariates associated with mortality^a^ in patients with lung high‐grade neuroendocrine tumor (HGNET)

	Lung cancer‐specific mortality				Overall mortality			
	Univariable analysis		Multivariable analysis^b^		Univariable analysis		Multivariable analysis^b^	
	HR (95% CI)	*P*‐value	HR (95% CI)	*P*‐value	HR (95% CI)	*P*‐value	HR (95% CI)	*P*‐value
PD‐L1 expression: positive (vs. negative)	0.23 (0.056–0.64)	0.0028	0.24 (0.058–0.67)	0.0039	0.28 (0.11–0.60)	0.0005	0.29 (0.11–0.61)	0.0006
p‐stage: II–IV (vs. I)	2.67 (1.37–5.59)	0.0034	3.22 (1.64–6.82)	0.0006	1.61 (0.97–2.75)	0.067	2.07 (1.23–3.56)	0.0062
Adjuvant chemotherapy: no (vs. yes)	1.88 (1.01–3.53)	0.048	2.55 (1.32–4.91)	0.0053	2.13 (1.29–3.54)	0.0033	2.64 (1.56–4.47)	0.0003
Neoadjuvant chemotherapy: yes (vs. no)	2.08 (0.99–4.05)	0.053	2.19 (1.02–4.39)	0.045	2.11 (1.15–3.67)	0.017	2.22 (1.19–3.93)	0.013
Smoking history: pack‐years < 20 (vs. ≥ 20)	1.59 (0.71–3.21)	0.24			1.30 (0.66–2.37)	0.43		
Histology: SCLC (vs. LCNEC)	1.21 (0.64–2.42)	0.57			1.13 (0.68–1.95)	0.64		
Age (years): ≥ 60 (vs. < 60)	1.20 (0.61–2.59)	0.61			1.73 (0.95–3.40)	0.074		
Lymphovascular invasion: positive (vs. negative)	1.16 (0.46–3.89)	0.77			1.05 (0.48–2.74)	0.91		
Gender: female (vs. male)	1.12 (0.53–2.76)	0.78			1.05 (0.53–1.91)	0.88		

We created missing categories for missing cases for each variable, if applicable. A backward stepwise elimination was carried out with *P *=* *0.05 as a threshold, to select variables for the final model. CI, confidence interval; HGNET, high‐grade neuroendocrine tumor; HR, hazard ratio; p‐stage, pathological stage; LCNEC, large cell neuroendocrine carcinoma; SCLC, small cell lung carcinoma.

a Cox proportional hazards regression models were used to calculate HR and 95% CI.

b Multivariable model initially included age (<60 vs. ≥60), gender (male vs. female), smoking history (≤20 vs. >20 pack‐years), pathological stage (p‐stage) (I vs. II–IV), neoadjuvant chemotherapy (yes vs. no), adjuvant chemotherapy (yes vs. no), histology (small cell lung carcinoma [SCLC] vs. large cell neuroendocrine carcinoma [LCNEC]), and lymphovascular invasion (positive vs. negative).

As secondary and exploratory analyses, we examined the prognostic associations of PD‐L1 positivity in SCLC and LCNEC patients using Cox regression models. The association of PD‐L1 positivity and other covariates with mortality in SCLC patients is shown in Table S1. In SCLC patients, PD‐L1 positivity was independently associated with lower lung cancer‐specific (multivariable HR = 0.11; 95% CI = 0.006–0.52; *P *=* *0.0020) and overall (multivariable HR = 0.19; 95% CI = 0.046–0.55; *P *=* *0.0010) mortality. Multivariable analysis was not possible in LCNEC patients because of low statistical power. In univariable analysis, PD‐L1 positivity showed a tendency toward lower lung cancer‐specific (univariable HR = 0.45; 95% CI = 0.070–1.69, *P *=* *0.26) and overall (univariable HR = 0.44; 95% CI = 0.10–1.30, *P *=* *0.15) mortality.

**Figure 3:**
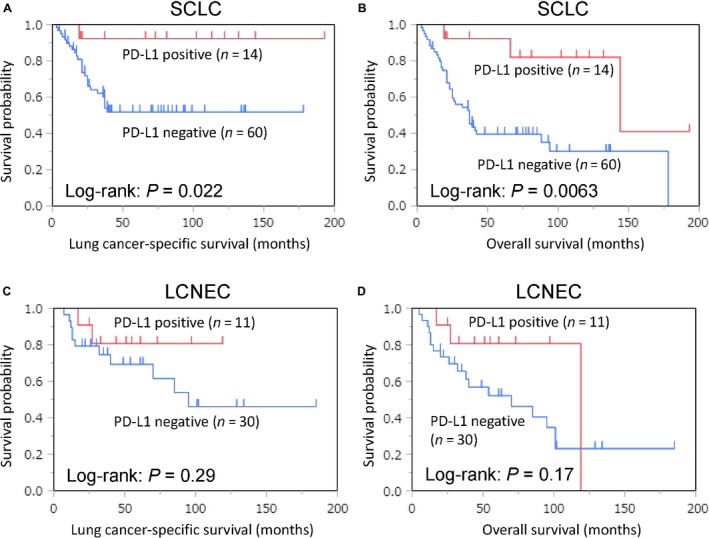
Kaplan–Meier curves for lung cancer‐specific (A, C) and overall survival (B, D) according to tumor programmed death‐ligand 1 (PD‐L1) expression status (positive vs. negative). (A, B) Small cell lung carcinoma (SCLC). (C, D) Large cell neuroendocrine carcinoma (LCNEC).

Using the Kaplan–Meier method and log‐rank test, we additionally examined the prognostic associations of tumor PD‐L1 positivity in SCLC and LCNEC patients (Fig. 3). PD‐L1 positivity was associated with lower lung cancer‐specific (log‐rank, *P *=* *0.022) and overall (log‐rank, *P *=* *0.0063) mortality in SCLC patients, whereas it showed a tendency of lower lung cancer‐specific (log‐rank, *P *=* *0.29) and overall (log‐rank, *P *=* *0.17) mortality in LCNEC patients.

## Discussion

We examined the association of tumor PD‐L1 expression with prognosis using 115 consecutive lung HGNET cases (74 SCLC and 41 LCNEC cases). PD‐L1 expression, being involved in immune modulation, is a potential predictive marker of the clinical response to anti‐PD‐1/PD‐L1 therapy. This therapy is effective in various cancers such as SCLC 6, 7, 8, 9, 10, 11, 12, 13, 14, 15. However, little is known about PD‐L1 expression in lung HGNET 20, 21, 22, which is characterized by heavy smoking history leading to a high mutation burden and more tumor antigens with increased immunogenicity 2, 3, 4, 5.

Little is known about the association of tumor PD‐L1 expression with HGNET prognosis and clinicopathological features. Only Ishii et al. examined the prognostic association of tumor PD‐L1 expression in 102 SCLC cases 20. They observed PD‐L1 positivity in 71.6% (73/102) of SCLC cases and showed that PD‐L1 positivity was independently associated with a favorable outcome, which is consistent with our study's results. Two studies examined the positivity of tumor PD‐L1 expression in SCLC 21, 22. Schultheis et al. examined 94 SCLC cases and did not observe tumor PD‐L1 positivity 21, whereas Komiya et al. examined 99 SCLC cases and observed tumor PD‐L1 positivity in 82.8% 22. We, Ishii et al., and Komiya et al. used a PD‐L1 cutoff level of 5%, whereas Schultheis et al. used the Allred system. The different scoring methods, different antibodies, different immunoreactivity in FFPE tissues, delayed/prolonged/inadequate fixation, inadequate fixatives, or pathological interpretive/analytical factors may explain this discrepancy. To our knowledge, this is the first study examining tumor PD‐L1 expression in HGNET including LCNEC and its association with prognosis. Therefore, our data are novel, informative, and translational.

The distinction between SCLC and LCNEC is challenging in terms of pathological diagnosis 31, 32, and both display similar clinicopathological characteristics such as high incidence in male and heavy smokers, high mitotic rates, neuroendocrine differentiation, and poor prognoses 33. Recently, SCLC and LCNEC have been classified into the same category of “neuroendocrine tumors” according to the 2015 WHO classification 24.

Small cell lung carcinoma shares genetic alterations with LCNEC 34, 35. A recent expansive genomics‐based classification study identified important similarities between SCLC and LCNEC regarding the transcriptome, particularly amplified and deleted regions and mutated genes. In that study, LCNEC and SCLC had significant mutations in *TP53*,* RB1*, and *EP300* and showed the same pattern of somatic copy number alterations 34.

Small cell lung carcinoma has been categorized into prognostically different subtypes, although SCLC is usually considered generally as a devastating cancer. By gene expression profiling, we previously identified two groups of HGNET (better‐ and worse‐prognosis subtypes) independent of the histopathological status (SCLC vs. LCNEC) 36. The better‐prognosis SCLC subtype was validated by gene expression profiling using independent samples; this subtype was immunohistochemically characterized by low neuroendocrine expression 37. Epigenetically, SCLC was categorized into a high‐CpG island methylator phenotype (CIMP) group with worse prognosis and a low‐CIMP group with better prognosis 38. Similarly, SCLC was categorized into high‐ and low‐DNA‐methylation groups; SCLC with high DNA methylation was associated with more aggressive disease behavior 39.

Large cell neuroendocrine carcinoma has been recently identified as a biologically heterogeneous tumor. Our gene expression profiling categorized LCNEC into two groups (better and worse prognosis) 36. Recently, a study using next‐generation sequencing identified SCLC‐like and NSCLC‐like LCNEC subtypes 40. The SCLC‐like LCNEC subtype showed higher proliferative activity, whereas the NSCLC‐like subtype had an overall genomic similarity with adenocarcinoma. LCNEC was also categorized by the immunohistochemical positivity of YAP1, the loss of which is a specific feature of HGNET 41. YAP1 loss was observed in 60% LCNEC patients and 98% SCLC patients; YAP1‐negative HGNET cases were more associated with chemosensitivity than YAP1‐positive cases 41.

We have shown that tumor PD‐L1 positivity was independently associated with lower mortality in lung HGNET (i.e., SCLC and LCNEC). The result in SCLC cases was similar to that obtained by Ishii et al. 20 The prognostic association of tumor PD‐L1 expression has not yet been previously demonstrated for LCNEC patients. SCLC and LCNEC are heterogeneous tumors with better and worse prognoses. Therefore, the prognostic association of PD‐L1 positivity with better prognosis in lung HGNET might be explained by the differential gene expression, different genetic mutations, high/low CIMP, DNA methylation degree, YAP1 positivity, SCLC‐like/NSCLC‐like phenotype, or high/low immunogenicity.

Our study has limitations. First, there is no standardized anti‐PD‐L1 antibody and scoring method of tumor PD‐L1 expression. For assessing PD‐L1 protein, several different clones are available. Nonetheless, we validated the specificity of a PD‐L1 antibody (clone: E1L3N) using a B7 subfamily cell array. Furthermore, we confirmed the protein and transcript expression of PD‐L1 in a SCLC cell line by conducting Western blotting and RT‐PCR analyses. Second, we utilized tissue microarrays to evaluate PD‐L1 expression in tumors. Because of intratumoral heterogeneity in lung HGNET, tumors with heterogeneous PD‐L1 expression can affect the results. This potential misclassification of tumors according to PD‐L1 expression would be randomly distributed; therefore, null results would have been obtained. Nonetheless, we have shown statistically significant associations. Furthermore, an experienced pulmonary pathologist (KI) chose each core site with a relatively large diameter (2 mm) in the most representative tumor histology. Therefore, core sites might not substantially affect the results. Third, we evaluated PD‐L1 expression only in tumor cells. PD‐L1 expression in tumor cells, tumor‐associated immune cells, or a combination of tumor and tumor‐associated immune cells might have biological or prognostic significance 6, 16, 21. Fourth, we used only surgical materials and not any inoperable cases, of which only biopsy materials were available. Because HGNET cases are often diagnosed at an advanced and inoperable stage 1, operable HGNET patients might have distinct features. Fifth, the total number of patients (*N *=* *115) was not sufficient, and the statistical power was therefore limited. Sixth, our dataset was retrospectively collected. Finally, this study may not be generalizable because we only enrolled Japanese patients in a single cancer hospital. Therefore, additional studies in other populations are needed.

In conclusion, we demonstrated the prevalence and clinicopathological characteristics of tumor PD‐L1 positivity in lung HGNET (i.e., SCLC and LCNEC) and the association of tumor PD‐L1 positivity with lower mortality in lung HGNET. This population‐based study provides useful information on PD‐L1 expression patterns in lung HGNET and may refine the role of PD‐L1 immunohistochemistry in clinical practice. Further studies with a larger sample size are warranted to confirm our findings.

## Conflict of Interest

Y.Y. is an employee of Daiichi Sankyo Co., Ltd. M.N. received research grants from Novartis Pharma Co., Ltd., Ono Pharma Co., Ltd., Chugai Pharma Co., Ltd., Pfizer Co., Ltd., Bristol‐Myers Squibb Co., Ltd., Eli Lilly Co., Ltd., Taiho Pharma Co., Ltd., Astellas Pharma Co., Ltd., and AstraZeneca Co., Ltd., and is a consultant for Novartis Pharma Co., Ltd., Ono Pharma Co., Ltd., Chugai Pharma Co., Ltd., Eli Lilly Co., Ltd., Taiho Pharma Co., Ltd., Pfizer Co., Ltd., and Daiichi Sankyo Co., Ltd. Y.I. received research grants from Daiichi Sankyo Co., Ltd., and Chugai Pharmaceutical Co. Ltd., and is a consultant for Fujirebio Inc. All other authors declare no conflicts of interest.

## Supporting information


**Figure S1.** Immunohistochemical evaluation of tumor membranous PD‐L1 expression in B7 subfamily cell array. Only PD‐L1 overexpressing cells (a) showed PD‐L1 positivity. Mock‐transfected cells (b) (nonspecific negative control) and other B7 families overexpressing cells (c–h) (specific negative controls) showed PD‐L1 negativity.
**Figure S2.** Western blot (a) and RT‐PCR (b) analyses for PD‐L1 using small cell lung carcinoma cell lines (STC‐1 and Lu135). Lu135 showed both PD‐L1 protein and transcript, whereas STC‐1 did not show PD‐L1 protein or transcript.

**Table S1.** PD‐L1 expression and other covariates associated with mortality* in patients with small cell lung carcinoma (SCLC).
