# Supplementary figures and images for: Relationship of tumor PD‐L1 (CD274) expression with lower mortality in lung high‐grade neuroendocrine tumor
Source: Cancer Med. 2017 Sep 18;6(10):2347–56. doi: 10.1002/cam4.1172 (PMC5633594; doi:10.1002/cam4.1172)

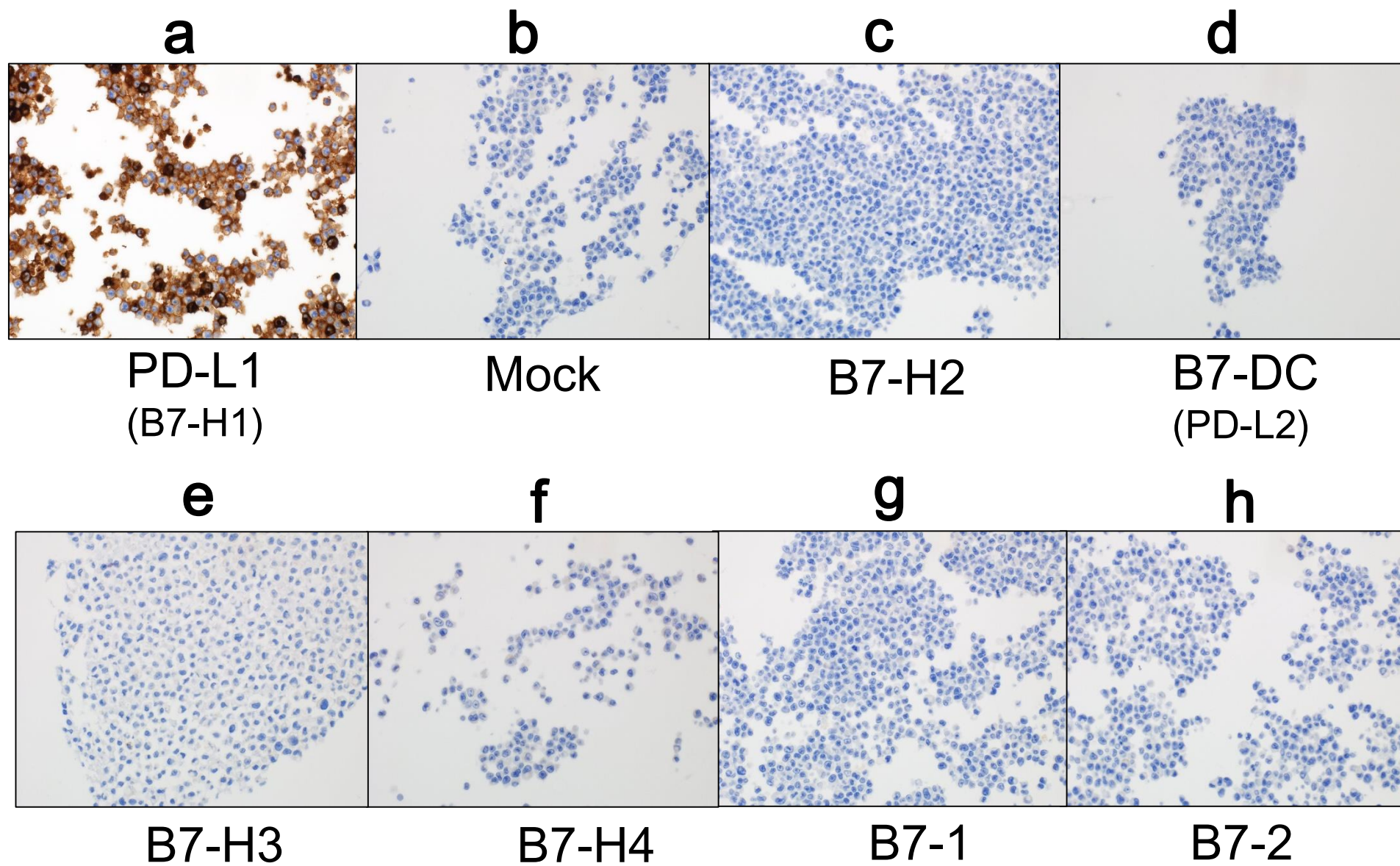

Supplementary Figure 1

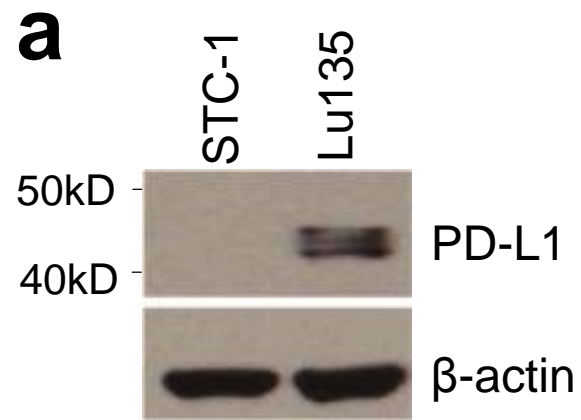

Western blot

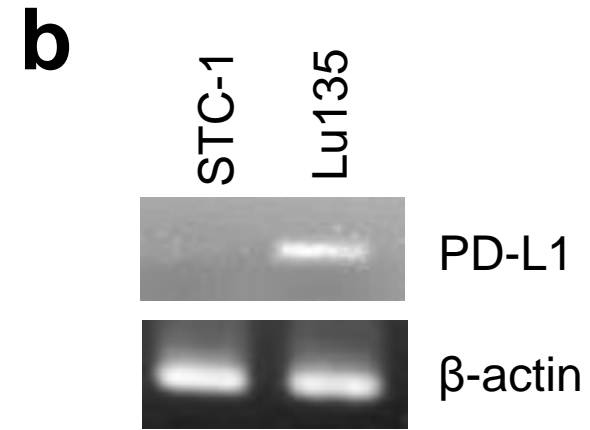

RT-PCR

Supplementary Figure 2

Supplement: Supplementary file 1 — Figure S1. Immunohistochemical evaluation of tumor membranous PD‐L1 expression in B7 subfamily cell array. Only PD‐L1 overexpressing cells (a) showed PD‐L1 positivity. Mock‐transfected cells (b) (nonspecific negative control) and other B7 families overexpressing cells (c–h) (specific negative controls) showed PD‐L1 negativity. Figure S2. Western blot (a) and RT‐PCR (b) analyses for PD‐L1 using small cell lung carcinoma cell lines (STC‐1 and Lu135). Lu135 showed both PD‐L1 protein and transcript, whereas STC‐1 did not show PD‐L1 protein or transcript. [file CAM4-6-2347-s001.pdf]
