# Supplementary material for: Relationship of tumor PD‐L1 (CD274) expression with lower mortality in lung high‐grade neuroendocrine tumor
Source: Cancer Med. 2017 Sep 18;6(10):2347–56. doi: 10.1002/cam4.1172 (PMC5633594; doi:10.1002/cam4.1172)
Supplement: Supplementary file 2 — Table S1. PD‐L1 expression and other covariates associated with mortality* in patients with small cell lung carcinoma (SCLC). [file CAM4-6-2347-s002.docx]

**Supplementary Table 1. PD-L1 expression and other covariates associated with mortality* in patients with small cell lung carcinoma (SCLC).**

|  | Lung cancer-specific mortality | | | |  | Overall mortality | | | |
| --- | --- | --- | --- | --- | --- | --- | --- | --- | --- |
|  | Univariable analysis | | Multivariable analysis** | |  | Univariable analysis | | Multivariable analysis** | |
|  | HR  (95% CI) | *P* value | HR  (95% CI) | *P* value |  | HR  (95% CI) | *P* value | HR  (95% CI) | *P* value |
| PD-L1 expression: positive  (vs. negative) | 0.14  (0.008-0.65) | 0.0070 | 0.11  (0.006-0.52) | 0.0020 |  | 0.22  (0.052-0.62) | 0.0022 | 0.19  (0.046-0.55) | 0.0010 |
| p-stage: II–IV  (vs. I) | 4.90  (1.88-16.7) | 0.0006 | 5.78  (2.22-19.7) | 0.0001 |  | 1.85  (0.96-3.79) | 0.068 | 2.33  (1.19-4.85) | 0.013 |
| Adjuvant chemotherapy: no  (vs. yes) | 2.28  (1.00-4.94) | 0.050 | 2.27  (1.00-4.91) | 0.051 |  | 2.59  (1.32-4.93) | 0.0066 | 2.62  (1.33-4.99) | 0.0062 |
| Neoadjuvant chemotherapy: yes  (vs. no) | 2.09  (0.94-4.49) | 0.069 |  |  |  | 2.21  (1.14-4.18) | 0.019 | 2.12  (1.08-4.07) | 0.030 |
| Smoking history: pack-years < 20  (vs. ≥ 20) | 2.84  (1.04-6.65) | 0.043 |  |  |  | 2.55  (1.09-5.31) | 0.033 |  |  |
| Lymphovascular invasion: positive  (vs. negative) | 3.52  (0.75-62.9) | 0.13 |  |  |  | 1.50  (0.54-6.24) | 0.48 |  |  |
| Age (years): ≥ 60  (vs. < 60) | 1.16  (0.51-2.96) | 0.73 |  |  |  | 1.38  (0.68-3.09) | 0.38 |  |  |
| Gender: female  (vs. male) | 1.05  (0.43-3.14) | 0.92 |  |  |  | 1.06  (0.45-2.20) | 0.88 |  |  |

(Continuing from the previous page.)

*Cox proportional hazards regression models were used to calculate HR and 95% CI.

**Multivariable model initially included age (< 60 vs. ≥ 60), gender (male vs. female), smoking history ($\leq$ 20 vs. > 20 pack-years), pathological stage (p-stage) (I vs. II‒IV), neoadjuvant chemotherapy (yes vs. no), adjuvant chemotherapy (yes vs. no), and lymphovascular invasion (positive vs. negative).

We created missing categories for missing cases for each variable, if applicable. A backward stepwise elimination was carried out with *P* = 0.05 as a threshold, to select variables for the final model.

CI, confidence interval; HR, hazard ratio; p-stage, pathological stage; SCLC, small cell lung carcinoma.
